# Economic Injury Level of the Psyllid, *Agonoscena pistaciae*, on Pistachio, *Pistacia vera* cv. Ohadi

**DOI:** 10.1673/031.009.4001

**Published:** 2009-06-12

**Authors:** Mohammad Reza Hassani, Gadir Nouri-Ganbalani, Hamzeh Izadi, Mahmoud Shojai, Mehdi Basirat

**Affiliations:** ^1^ Department of Entomology, Science and Research Branch, Islamic Azad University, Tehran, Iran; ^2^ Mohaghegh Ardabili University, Ardabil, Iran; ^3^ Vali-e-Asr University, Rafsanjan, Iran; ^4^ Pistachio Research Institute, Rafsanjan, Iran

**Keywords:** pistachio psylla, economic damage, yield loss, decision making

## Abstract

The pistachio psylla, *Agonoscena pistaciae* Burckhardt and Lauterer (Hemiptera: Psyllidae) is a major pest of pistachio trees, *Pistacia vera* L. (Sapindalis: Anacardiaceae) throughout pistachio-producing regions in Iran. Different density levels of *A. pistaciae* nymphs were maintained on pistachio trees by different insecticide dosages to evaluate the relationship between nymph density and yield loss (weight of 1000 nuts). Psylla nymph densities were monitored weekly by counting nymphs on pistachio terminal leaflets. There was a significant reduction in weight of 1000 nuts as seasonal averages of nymphs increased. Regression analysis was used to determine the relationship between nymph density and weight of 1000 nuts. The economic injury levels varied as a function of market values, management costs, insecticide efficiency and yield loss rate and ranged from 7.7 to 30.7 nymphal days per terminal leaflet, based on weight of 1000 nuts.

## Introduction

The pistachio, *Pistacia vera* L. (Sapindalis: Anacardiaceae), is one of the most important horticultural products in Iran. The pistachio psylla, *Agonoscena pistaciae* Burckhardt and Lauterer (Hemiptera: Psyllidae) is a key pest of pistachio trees throughout the pistachio-producing regions of Iran. Both nymphs and adults suck sap from leaves and produce large amounts of honeydew. Direct feeding causes reduced plant vigor, defoliation, stunting, poor yield and bud drop (Samih et al. 2005). The control of this pest has been based on insecticides with no attention to the pest density and economic damage. The economic injury level is an important component of cost-benefit in integrated pest management (IPM) program and is a useful tool for decision-making in application of pesticides (Shipp et al. 2000). The EIL concept represents a theoretical foundation for IPM, because it provides information on how many pests can cause economic damage and how many are tolerable (Peterson and Higley 2002). Stern et al. (1959) defined EIL as “The lowest population density pest that will cause economic damage or the amount of pest injury which will justify the control costs”. Pedigo et al. (1986) showed that EIL is actually a level of injury indexed by pest numbers. The parameters for calculating EILs include the cost of management tactics, market values, yield loss rate and efficiency of management tactics (Higley and Pedigo 1996).

There is no information on the relationship between *A. pistaciae* nymph density and pistachio yield loss in Iran. The objective of this study was to determine the yield loss function in pistachio trees, *P. vera* L. cv. Ohadi based on *A. pistaciae* nymph density. This information was used to determine the EILs for *A. pistaciae* that can be used to reduce costs and insecticide application through judicious use of chemical control.

## Materials and Methods

The experiment was conducted in a pistachio orchard at Rafsanjan, Iran in 2007. Rafsanjan is one of the main pistachio producing areas of Iran and the world (Razavi 2005). The experiment was conducted on *P. vera* cv. Ohadi, the most common cultivar in this region. Treatments were arranged in a randomized complete block design with 5 replicates (the replicates were single trees). Thirty trees of similar age were selected in a commercial orchard. The trees were irrigated and fertilized according to standard recommendations for the region and the age and crop loads of the trees horticultural care were similar. During this research, the trees were not caged and their yields were similar. At the beginning of spring, mid-April to time of harvest, in late September, different density levels of psylla nymphs were maintained on pistachio trees by applying different low concentration dosages of the insecticide amitraz (Mitac®) to the whole tree in the spring. To achieve the different nymph densities 0.2, 0.4, 0.6, 0.8 ml/l of amitraz 20 EC and 1 ml/l were applied and for the highest density no insecticide was applied. These rates of insecticide applied achieved different *A. pistaciae* densities. The density of nymphs was monitored weekly by recording nymphs per terminal leaflet by visual observation. In each of the 30 trees, 8 terminal leaflets were randomly selected from the middle height of each tree and the number of nymphs was recorded per terminal leaflet. The control group was maintained at a pest level close to zero by treating it with a normal dosage of amitraz 20 EC, at a concentration of 1 ml/l. At the end of the pistachio growing season, the yield of each tree was determined and a sample of 1000 nuts was taken and its weight recorded to provide an estimate of yield loss. Analysis of variance (ANOVA) was used (SAS Institute 1987) to compare the differences, and F-tests were conducted for seasonal averages of nymph density and weight of 1000 nuts. Regression analysis (SAS Institute 1987) was used to determine the relationship between nymph density and weight of 1000 nuts to calculate EILs. The EILs was estimated by using the Pedigo et al. (1986) equation as follows:

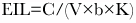

Where C is the control costs (insecticide cost + insecticide application cost) per production unit ($/ha), V is the market value per production unit ($/kg), b is the slope of the regression of nymph numbers on yield loss, and K is the reduction of injury due to treatment (proportion 0.8 for 80% mortality to 1 for no loss). Among the above characters, market value is the most obvious uncertain variable to estimate EIL because pest management action is required several weeks before crop harvest. Thus, it is necessary to estimate the market value at the time of sale. The management cost is more certain because of known values for insecticide and application costs at close to the time of management action (Peterson and Hunt 2003).

The basic steps to calculate EIL are:

- Estimate yield loss (b)
- Determine the gain threshold
- Determine yield loss that can avoided by applying a management tactic
- Calculate EIL as:

EIL=gain threshold/ (yield loss rate per insect×amount of loss avoided)

To calculate EILs, the gain threshold was calculated as the amount yield must be increased to compensate for the cost of management (Stone and Pedigo, 1972). This has been used to express the beginning of economic damage, and as a measure of marketable yield per special land area (Pedigo 2002).

**Figure 1.**
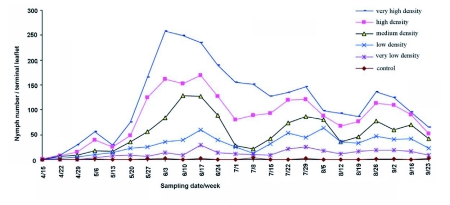
Mean numbers of *Agonoscena pistaciae* nymphs in different treatments on pistachio, *Pistacia vera* cv. Ohadi in Rafsanjan, Iran, 2006.

gain threshold (kg/ha)=management cost ($/ha)/market value ($/kg)

To investigate the relationship between pistachio nymph density and weight of 1000 nuts, the nymphal days (ND) index (Burts 1988) was used, calculated as follows:

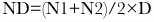



Where N1 is nymphs per leaf in a given count, N2 is nymphs per leaf in the count following N1, and D is days between N1 and N2. The sampling intervals were seven days.

## Results and Discussion

Seasonal averages of *A. pistaciae* nymph densities for different treatments are presented in Figure 1. Seasonal averages of nymph densities differed between insecticide treatments during the pistachio growing season. Population densities increased in spring, with a maximum in mid-June. At this time, pistachio trees can be severely damaged by high populations at the beginning of embryonic development. The psylla numbers decreased in summer.

**Figure 2.**
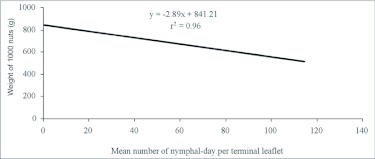
Linear regression of *Agonoscena pistaciae* nymph number and weight of 1000 nuts on *Pistacia vera* cv. Ohadi, Rafsanjan, Iran, 2006.

The infestation levels of psylla nymphs differed among treatments, allowing testing of the effect of nymphs on yield loss. There was a significant decrease in weight of 1000 nuts as seasonal averages of nymph numbers increased. The weight of 1000 nuts was significantly affected by the number of nymphs (F= 22.71; df = 5, 24; P< 0.0001). A regression equation was used to describe the relationship between nymph density and weight of 1000 nuts; y = -2.89x + 841.21, r^2^ = 0.96 (Figure 2), where y is weight of 1000 nuts and × is the number of nymphal days per terminal leaflet. The linear relationship between the weight of 1000 nuts and seasonal averages of nymph number indicates that relatively large yield loss can occur with increased nymph numbers.

Based on the market values of $3–4 per kg, and management costs of $80–240 per ha, the gain threshold was 20–80 kg/ha. This means 20–80 kg yields must be saved per ha to pay the cost of treatment. In other words, insecticide application would need to save at least 20–80 kg per ha to justify the activity.

Finally, the EILs were calculated as the number of *A. pistaciae* nymphs required to cause the critical yield loss from the predictive model. However, pistachio price and management cost, including insecticide and application cost, change each year. Therefore, to generalize EIL for different years the calculation was based on the range of market values and management costs. The calculated range of EILs of pistachio psylla nymphs on pistachio cv. Ohadi in Rafsanjan, Iran ranged from 7.7 to 30.7 nymphal days per terminal leaflet (Table 1).

**Table 1.**
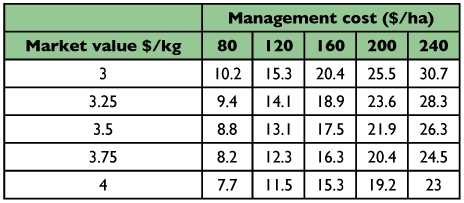
Economic injury levels (nymphal days per terminal leaflet) for Agonoscena pistaciae nymphs on Pistacia vera cv. Ohadi, Rafsanjan, Iran.

The EILs vary as a function of market values, insecticide and application costs and efficiency of insecticide. Therefore, EIL must be recalculated when these factors change. Conceptually, the EIL is a cost-benefit equation in which the costs are balanced with benefits. The EIL is an important conceptual and practical tool for decision-making in IPM programs (Higley and Pedigo 1996). The EIL values propose the psylla nymph densities that cause sufficient damage to justify treatment costs. Therefore, it is suggested that pistachio producers use insecticides only when the nymph density reaches the EIL. This information may be considered by plantation managers as a first-order guideline for pest management decision-making to reduce costs of insecticide use, and so increase profitability and conserve environmental quality. The relationship between psylla nymph density and weight of 1000 nuts obtained in this study provides basic information to estimate EIL, based on market value, management cost and insecticide efficiency.

## **Abbreviations**

EIL: economic injury level
